# The British Journals, &c.: Animal Chemistry and Pharmacy

**Published:** 1842-04

**Authors:** 


### ANIMAL CHEMISTRY AND PHARMACY.

EXPERIMENTS ON ASAFŒTIDA. For determining the quantity of sulphur in asafœtida a Florence flask, surmounted by a couple of funnels, answers perfectly, as the quantity of nitrous acid always present in the upper space prevents the possibility of any of the sulphur being dissipated in the form of sulphurous acid gas. After the whole of the asafœtida has been apparently acidified into a transparent liquid by a lengthened ebullition, it deserves remark, that no trace of sulphuric acid will be found in it by the solution of any barytic salt; and that to make this test effective the liquid must be evaporated nearly to dryness, and then treated with distilled water. On filtering this solution, and pouring into it the barytic test, a copious cloud immediately ensues. From one hundred grains of good asafœtida thus oxygenated, sulphate of baryta will be obtained equivalent to two grains of sulphur. This quantity is the mean result of a great many experiments upon different samples of the drug. I shall endeavour, in a future communication to the Society, to account for the singular fact, why the acidified sulphur does not in the first instance affect solutions of baryta.

Asafœtida occurs in commerce in very different states of purity and composition. A specimen in *tears*, furnished by Mr. Herring, had a specific gravity so low as 1·25 (which is that of fine cochineal), and it left after incineration in a platinum capsule, only five per cent. of earthy and alkaline matter, chiefly phosphate of lime, with a little carbonate of potassa. Good common asafœtida, milky-white, semi-hard, has a specific gravity of 1·31, and affords about twelve per cent. of incombustible ashes, consisting of silica, carbonate and phosphate of lime, with a trace of potassa. Ordinary massive asafœtida has a specific gravity of 1·35. It affords twenty-five per cent. of incombustible ashes, consisting in a great measure of siliceous and calcareous gravel, from the soil round the root of the plants which yielded the asafœtida.

Pure asafœtida consists of about 44½ per cent. of resin and 2 of oil. The sulphur is contained in the resin, and has probably a share in the production of the offensive fumes most irritating to the eyes, which are exhaled from the resin at and above its fusing point of 243° F.—Dr. URE, *Pharmaceutical Journal*, No. ix. *March*, 1, 1842.

---

ARTIFICIAL HARROWGATE SALTS. Where the genuine Harrowgate water cannot well be procured, the following will be found a good substitute:

℞ Sulphatis potassæ cum Sulphure (Ph. Ed.) .. ʒj.

Potassæ bitartratis ...................... ʒss.

Magnes. sulphat ......................... ʒvj.

Aquæ destillat .............................. lbij.

Solve.—Capiat dimidium p. r. n.

The above is sufficient for a quart, and ought to be taken early in the morning before breakfast, and be followed by a walk, to produce the desired effect.

The artificial Harrowgate salts are very much employed, and not unfrequently by those who drink the genuine water, for the purpose of increasing its aperient power. The salts may be made as follows:

℞ Sulph. potass. cum sulphure .............. ʒvj

Potass. bitartratis ........................ ℥j

Pulv. magn. sulph. ......................... ℥vj

M. bene.

The usual dose of the above is one teaspoonful in a small tumbler full of tepid water, early in the morning.—Mr. JOHN MACKAY, *Pharmaceutical Journal*, No. vii. *Jan.* 1, 1842.

